# Does respiratory health contribute to the effects of long-term air pollution exposure on cardiovascular mortality?

**DOI:** 10.1186/1465-9921-8-20

**Published:** 2007-03-07

**Authors:** Tamara Schikowski, Dorothea Sugiri, Ulrich Ranft, Ulrike Gehring, Joachim Heinrich, H-Erich Wichmann, Ursula Krämer

**Affiliations:** 1 Institut für Umweltmedizinische Forschung (IUF) at the Heinrich-Heine-University Düsseldorf, Auf'm Hennekamp50, 40225 Düsseldorf, Germany; 2 GSF – National Research Center for Environment and Health, Institute of Epidemiology, Ingolstädter Landstrasse 1, 85764 Neuherberg, Germany; 3 Ludwig-Maximilians-University of Munich, Institute of Medical Data Management, Biometrics and Epidemiology, Chair of Epidemiology, Geschwister-Scholl Platz 1, 80539 Munich, Germany; 4 Utrecht University, Institute for Risk Assessment Sciences, P.O. Box 80.176, NL-3508 TD Utrecht, The Netherlands

## Abstract

**Background:**

There is growing epidemiological evidence that short-term and long-term exposure to high levels of air pollution may increase cardiovascular morbidity and mortality. In addition, epidemiological studies have shown an association between air pollution exposure and respiratory health. To what extent the association between cardiovascular mortality and air pollution is driven by the impact of air pollution on respiratory health is unknown. The aim of this study was to investigate whether respiratory health at baseline contributes to the effects of long-term exposure to high levels of air pollution on cardiovascular mortality in a cohort of elderly women.

**Method:**

We analyzed data from 4750 women, aged 55 at the baseline investigation in the years 1985–1994. 2593 of these women had their lung function tested by spirometry. Respiratory diseases and symptoms were asked by questionnaire. Ambient air pollution exposure was assessed by the concentrations of NO_2 _and total suspended particles at fixed monitoring sites and by the distance of residency to a major road. A mortality follow-up of these women was conducted between 2001 and 2003. For the statistical analysis, Cox' regression was used.

**Results:**

Women with impaired lung function or pre-existing respiratory diseases had a higher risk of dying from cardiovascular causes. The impact of impaired lung function declined over time. The risk ratio (RR) of women with forced expiratory volume in one second (FEV_1_) of less than 80% predicted to die from cardiovascular causes was RR = 3.79 (95%CI: 1.64–8.74) at 5 years survival time and RR = 1.35 (95%CI: 0.66–2.77) at 12 years. The association between air pollution levels and cardiovascular death rate was strong and statistically significant. However, this association did only change marginally when including indicators of respiratory health into the regression analysis. Furthermore, no interaction between air pollution and respiratory health on cardiovascular mortality indicating a higher risk of those with impaired respiratory health could be detected.

**Conclusion:**

Respiratory health is a predictor for cardiovascular mortality. In women followed about 15 years after the baseline investigation at age 55 years long-term air pollution exposure and impaired respiratory health were independently associated with increased cardiovascular mortality.

## Background

There is growing evidence that short and long-term exposure to high levels of air pollution may increase cardiovascular morbidity and mortality [1-5]. In addition, epidemiological studies have shown an association between increased levels of air pollution and exacerbations of airways diseases [6] or impairments of lung function [7]. There is also support for a link between respiratory health and cardiovascular mortality [8-10]. To what extent the association between cardiovascular mortality and air pollution is driven by the impact of air pollution on respiratory health is unknown. It is hypothesised that pulmonary inflammation induced through harmful particles may cause the release of mediators that increase blood coagulation [11,12]. Other studies have shown that increased blood coagulability or viscosity is a risk factor for cardiovascular mortality [13]. However, other mechanisms not related to respiratory health including systemic inflammation, accelerated atherosclerosis and altered cardiac autonomic function may also be responsible for the effect of particle exposure on cardiovascular mortality [4].

Studies have shown that people with pre-existing respiratory disease have a higher risk of dying from cardiovascular causes due to short-time variations in air pollution exposure [14-17]. Whether people with pre-existing respiratory disease have a higher risk of dying from cardiovascular disease after long-term air pollution exposure is not clear. We have shown that high levels of air pollution were associated with a reduction in lung function, impaired respiratory health and chronic obstructive lung disease [18] in women aged 55 years from the Ruhr Area in 1985–1994. We also showed that these levels of air pollution increased the risk of mortality in the same group of women during a follow-up until 2002/2003 [19].

In this presented study, we investigated whether respiratory health at baseline contributes to the effects of long-term exposure to high levels of air pollution on cardiovascular mortality in this cohort of elderly women. Indicators of respiratory health at baseline investigation were chronic bronchitis and respiratory symptoms as well as lung function measures. In compliance with the study objective, the following questions were to be answered:

(1) Is impaired respiratory health a risk factor for cardiovascular mortality?

(2) Alongside long-term air pollution exposure, is impaired respiratory health an independent risk factor for cardiovascular mortality?

(3) Is there a difference in pollution induced cardiovascular mortality in people with and without impaired respiratory health?

## Method

### Study population

The SALIA cohort (***S***tudy on the influence of ***A***ir pollution on ***L***ung function, ***I***nflammation and ***A***ging) was initiated as part of the Environmental Health Surveys introduced by the North Rhine Westphalia government in the mid 1980s, focusing on the effect of air pollution on respiratory health in women and children. Consecutive cross-sectional studies were performed between 1985 and 1994 in the Ruhr area and two rural towns as reference areas. The study population comprised 4874 women aged 55 at the time of entering the study who were living in pre-defined residential areas and willing to participate. In the years specified, the study areas included Dortmund (1985, 1990), Duisburg (1990), Essen (1990), Gelsenkirchen (1986, 1990) and Herne (1986) which represent a range of high-polluted areas. The two rural towns, Borken (1985, 1986, 1987, 1990, 1993, and 1994) and Dülmen (1986) were chosen as reference areas. About every second responder was invited to have her pulmonary function tested, exceptions were Dortmund in 1990 where no lung function measurements were performed and Borken in 1993/94 where all women were invited to participate (N = 2593).

### Follow-up study

The follow-up study was conducted by the Institute of Epidemiology (GSF Munich) between January 2002 and May 2003. All women were followed for the cause of specific mortality. Causes of death were obtained from official death certificates and were coded according to the International Classification of Diseases, Ninth Revision (ICD-9). Mortality for all causes of death and cardiovascular (ICD9-400-440) causes were recorded. The analysis was restricted to 4750 from the 4874 women whose complete information was available from the baseline investigation and who could be followed-up in 2002–2003. Women who moved during the follow-up period and who were lost for the follow-up after moving were judged censored at the time of movement. Otherwise, survival time was censored at the time of follow-up or the time of death from causes other than cardiovascular. The cause of death is known for 399 women. The analysis presented focuses on cardiovascular mortality.

### Assessment of risk factors for respiratory health and cardiovascular mortality

Baseline co-morbidities and potential risk factors such as smoking and the level of education were assessed by a self-administered questionnaire. All returned questionnaires were checked by the investigating physician. We grouped the women according to their reported smoking habits: never smoker without environmental tobacco smoke (ETS), passive-smoker (ETS at home and/or work place), past smoker and current smoker (<15 pack years; 15–30 pack years and >= 30 pack years). Current smokers with missing information about the numbers of cigarettes smoked were assigned to smokers with > = 30 pack years. These variables were used to control for confounding. Their socio-economic status was determined by categorizing the women into three levels of education using the highest school level completed by either the women or her husband as low (< 10 years), medium (= 10 years) or high (> 10 years).

### Assessment of respiratory health by questionnaire

Identical standardized self-administered questions were used during the entire screening period from 1985–1994. The questionnaire included questions about impaired respiratory health. The following questions were used to describe frequent cough with or without phlegm production:

Do you usually cough in the morning, when you get up or during the day?

If yes: Do you produce phlegm when you have this cough?

These questions are part of the classical definition of chronic bronchitis [20]. We further asked:

Do you have a physician's diagnosis of chronic bronchitis?

### Assessment of respiratory health by pulmonary function

Spirometry was conducted using a Vica Test 4 spirometer (Mijhardt, Rotterdam, The Netherlands). All measuring instruments were calibrated prior to each session. At least two acceptable spirograms were obtained from a minimum of four forced expirations. A trained technician identified the best single spirogram. All staff was specifically trained and the same measuring device was used throughout the study. In our analysis, we used the forced expiratory volume in one-second (FEV_1_) and the forced vital capacity (FVC). Linear regression models were used to predict the lung function parameter FEV_1 _and FVC based on age, height, race and sex. We used the equations which are recommended by the American Thoracic Society [21]. The prediction equations for creating reference values for these women were:

FEV_1 _^predicted ^= 0.433-0.0036*age-0.00019*age^2^+0.000115*height^2^

FVC^predicted ^= -0.356+0.0187*age*0.00038*age^2^*0.000148*height^2^

We defined impaired lung function by using FEV_1 _< 80% and FVC < 80% of the predicted value of each parameter. These cut-offs were also used in the re-analysis of the Harvard Six City Study [5]. To verify that these reference equations were suitable for our study collective, we applied them to the women living in the rural areas (reference areas). It turned out that the reference equations fitted very well the lung function values of these women, i.e. 5% of these women had lung function values below the 80% cut-offs.

### Assessment of air pollution exposure

We obtained the air pollution measurements data from 8 monitoring stations maintained by the State Environment Agency of North-Rhine Westphalia. In each city concentrations of ambient air pollutants were measured at fixed monitoring sites representing urban background levels. The monitoring stations are located in an 8 km grid throughout the women's residential areas. However, the air pollution data from Borken and Dülmen are incomplete, because continuous measurements in this region started in 1990. For the years proceeding 1990, the data were imputed by using measurements (1981–2000) from 15 monitoring stations in the Ruhr area assuming similar trends. Estimated 'average' differences were added to the levels measured in 1990 to 1991 for the imputation of air pollution concentrations in the years before 1990. The estimated average differences were 1.02 μg/m^3 ^per year for NO_2 _and 1.36 μg/m^3^per year for PM_10. _More details can be found elsewhere [19].

To estimate the long-term air pollution exposure we used five-year means of measurements done before the investigation. The concentrations of nitrogen dioxide (NO_2_) were measured half-hourly by means of chemo-luminescence. Total suspended particles (TSP) were gathered with a low volume sampler (air flow: 1 m^3^/h) and measured using beta-ray absorption. For reasons of comparability with studies based on PM_10 _measurements (particulate matter with aerodynamic diameters less than 10 μm), we estimated the corresponding PM_10 _values by multiplying the TSP measurements with a conversion factor of 0.71. Details for justification of this conversion factor can be found elsewhere [19]. We further used geographic information system (GIS) software Arc GIS 9.0 (ESRI Redlands, Cato) to calculate the distance of the residential address to the nearest major road with more than 10,000 cars/day. A distance of 50 m to the nearest major road was used as cut-off to reflect small-scale spatial variations in traffic related exposure. Traffic counts were provided by the North Rhine Westphalia State Environment Agency (LUA NRW).

### Statistical methods

Cox' proportional hazard regression model was used to analyze the association between cardiovascular mortality, air pollution exposure and respiratory health. Following the study questions, three analysis steps were done:

First, we investigated whether cardiovascular death was associated with impaired respiratory health. The assumption of proportional hazard was tested by introducing a time-dependent covariate into the Cox' model [22]. This new variable was defined as the product of the logarithm of survival time with the binary variable characterising impaired respiratory health. The proportionality assumption was rejected when the regression coefficient of this covariate was significantly (p < 0.1) different from the null value. We presented relative risks of cardiovascular death due to respiratory health impairment at two survival times (5 years (60 month) and 12 years (144 month)) which correspond roughly to the 25^th ^and 75^th ^percentile of the survival time distribution of those who died in the study group.

Second, the risk ratios of cardiovascular mortality for each air pollution indicator were estimated adjusted for potential confounders (model (a)). Educational level and smoking behaviour had already been identified as relevant confounders in our previous paper [19]. Then, respiratory health indicators were additionally considered in the Cox' regression analysis (model (b)). If the assumption of hazard proportionality for the respiratory health strata was not met (result of step one) then a stratified analysis was done and, if no interaction between respiratory health and air pollution exposure had to be taken into account (if otherwise see step three), common risk ratio estimates of the strata were given. No or negligible differences between the estimated risk ratios for air pollution exposure between model (a) and model (b) indicate that respiratory health is an independent risk factor for cardiovascular mortality alongside air pollution exposure.

Third, it was determined whether the relative risks for air pollution associated cardiovascular mortality were different in strata defined by respiratory health. Because of the small power of interaction tests a p-value of 0.3 or less was chosen as indication for interaction. If the p-value was less, then no combined estimates but estimates for both strata are given separately.

Risk ratio estimates of continuous exposure measures refer to unit steps as chosen in [18,19], i.e. 16 μg/m^3 ^and 7 μg/m^3 ^for NO_2 _and PM_10_, respectively.

Survival times in subgroups defined by respiratory health indicators were graphically depicted by Kaplan-Meier curves with 95 percent confidence limits.

All analyses were conducted with the statistical software SAS. For Cox' regression analysis, we used the procedure PHREG of SAS version 9.1 for windows (SAS Institute Cary, NC).

## Results

### Description of the study participants

In total, 4750 women were in the study, and a percentage of 54.5% underwent lung function testing. Distribution characteristics of the whole study group and, separately, of the sub-group with lung function measures are summarised with respect to respiratory health, mortality and other socio-demographic indicators in table 1. Due to the study design the women who had their lung function tested lived to a larger extent in rural areas and related to that they were to some extent healthier and smoked less than those in the whole study group. Again, because of the design, air pollution exposure in the sub-group with spirometry was slightly lower than in the whole study group (table 2).

**Table 1:** Characteristics of impaired respiratory health, mortality and socio-demographics of a cohort of women aged 55 years at baseline investigation

	***Whole study group N = 4750***		***Study group with spirometry N = 2580***	
	
	***n/N***	***%***	***n/N***	***%***
FEV_1 _<80% of predicted value	--	--	409/2577	15.9
FVC <80% of predicted value	--	--	526/2571	20.5
Chronic Bronchitis by physician diagnose	442/4642	9.5	211/2525	8.4
Frequent cough with phlegm production	518/4700	11.0	266/2554	10.4
Frequent cough	1063/4724	22.5	560/2568	21.8
All cause death	399/4750	8.4	183/2580	7.1
Cardiovascular death	127/4750	2.7	53/2580	2.1
Never smoker without ETS	1779/4750	37.5	1191/2577	46.2
Never smoker with ETS	1494/4750	31.5	829/2577	32.2
Ex-smoker	377/4750	7.9	201/2577	7.8
Current smoker with < 15 pack years	270/4750	5.7	136/2577	5.3
Current smoker with 15–30 pack years	284/4755	6.0	137/2577	5.3
Current smoker > 30 pack years	224/4755	4.7	83/2577	3.2
Smoking behaviour unknown	322/4750	6.8	143/2577	5.5
Living in rural area	1681/4750	35.4	1315/2580	51.0
Less then 10 y school	1400/4695	29.8	685/2574	26.6
At least 10 y school	2243/4695	47.8	1253/2574	48.7
More then 10 y school	1052/4695	22.4	636/2574	24.7
	***N***	***Mean/SD***	***N***	***Mean/SD***

Age [years]	4748	54.5/0.6	2576	54.5/0.7

Abbreviations:

FEV_1_: Forced expiratory volume in 1 second; FVC: Forced vital capacity; SD: Standard deviation

**Table 2:** Distribution of women depending on their ambient air pollution exposure (5 year mean values prior to baseline investigation) and traffic exposure indicated as percentiles

	***Mean/Percentage***	***P0***	***P25***	***P50***	***P75***	***P100***
***Whole study group (N = 4750)***						
NO_2 _[μg/m^3^]	39	22	25	46	49	55
PM_10 _[μg/m^3^]	48	39	43	47	53	56
<50 m distance to major road (>10,000 cars/day)	8.5 %	--	--	--	--	--
***Study group with spirometry (N = 2580)***						
NO_2 _[μg/m^3^]	36	22	24	27	50	53
PM_10 _[μg/m^3^]	47	39	43	47	52	54
<50 m distance to major road (>10,000 cars/day)	7.6 %	--	--	--	--	--

Abbreviations:

Px: x^th ^percentile; NO_2: _Nitrogen dioxide; PM_10_: Particulate matter with aerodynamic diameter of ≤ 10 μm, calculated as PM_10 _= 0.71*TSP; TSP: Total suspended particles

### Respiratory health and cardiovascular mortality

**Figure 1:**
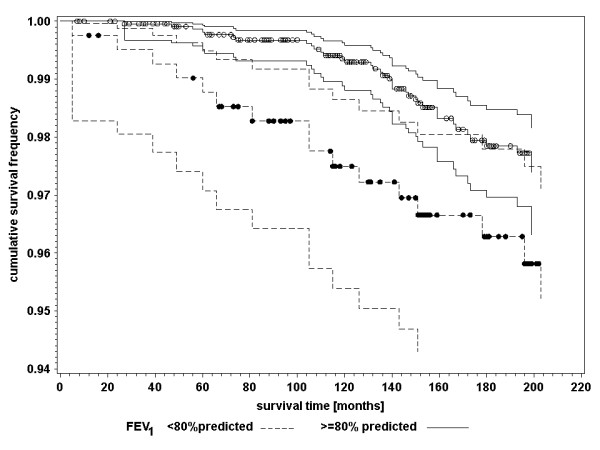
Kaplan-Meier survival curves with 95 percent confidence limits of cardiovascular mortality for women aged 55 years at baseline investigation with FEV_1 _< 80% predicted and FEV_1 _≥ 80% predicted; dots indicating censored events. *Abbreviations: *FEV_1_: Forced expiratory volume in 1 second.

**Figure 2:**
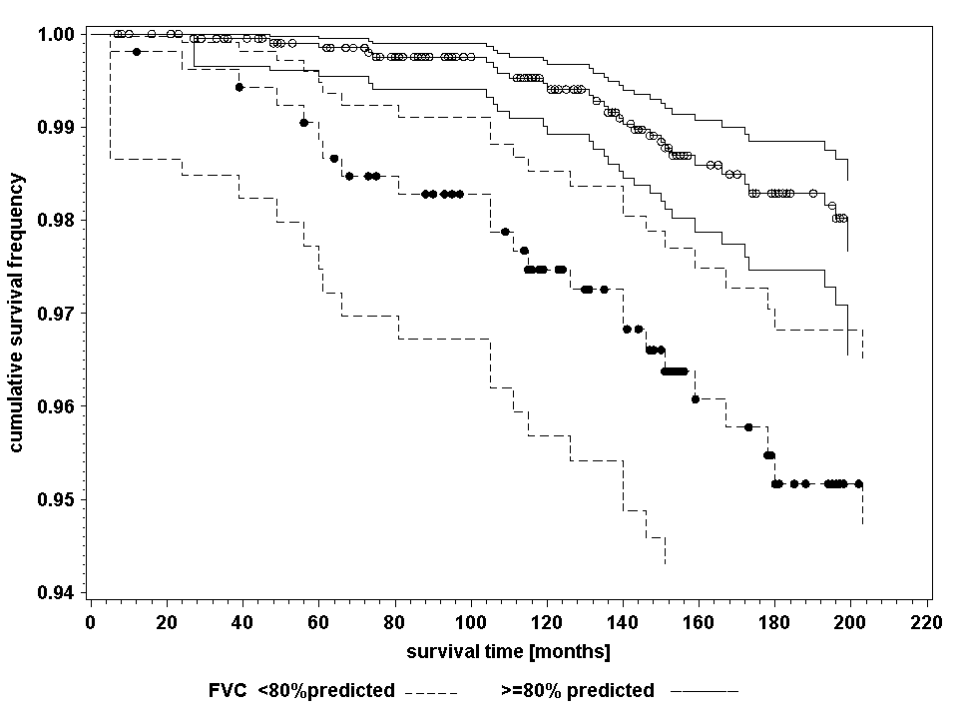
Kaplan-Meier survival curves with 95 percent confidence limits of cardiovascular mortality for women aged 55 years at baseline investigation with FVC < 80% predicted and FVC ≥ 80% predicted; dots indicating censored events. *Abbreviations: *FVC: Forced vital capacity

In table 3, crude risk ratios (RR_c_) demonstrate that cardiovascular death was associated with impaired respiratory health and unfavourable lung function values. The association between cardiovascular mortality and impaired respiratory health defined by diagnosis and symptoms demonstrated a different time pattern than that defined by lung function measurements. The association of the diagnosis of chronic bronchitis with cardiovascular mortality did not change over time: Women with the diagnosis of chronic bronchitis had an increased risk ratio of dying from cardiovascular causes at 60 months survival time (RR_c _= 1.53; 95% CI: 0.83–2.79) and at 144 months survival time (RR_c _= 1.65; 95% CI: 0.93–2.95). Similar results were found for frequent cough with phlegm production. The impact of impaired lung function at age 55 years on cardiovascular mortality however declined over time. Figure 1 and 2 show the survival curves of women with and without impaired FEV_1 _and FVC. The proportionality assumption is not valid. Interaction with survival time was significant for both lung function indicators (table 3). The risk of women with impaired lung function at age 55 years to die from cardiovascular causes at the age of 60 years, was 3.8 to 5.0 times higher than the risk of women without pathological findings of the lung function. The risk ratio at the age of 67 years declined near the null value (table 3).

**Table 3:** Crude risk ratios (RR_c_) and 95% confidence interval (95% CI) of cardiovascular mortality for impaired respiratory health and lung function indicators at 5 and at 12 years of survival time and p-value for interaction with baseline, results of Cox' regression analysis.

***Respiratory symptoms and lung function***	***RR***_*c*_, ***95% CI at 5 years***	***RR***_*c*_, ***95% CI at 12 years***	***p-value for interaction with baseline***
Chronic Bronchitis by physician diagnose	1.53 0.83–2.79	1.65 0.93–2.95	0.7986
Frequent cough with phlegm production	1.34 0.71–2.51	1.65 0.94–2.89	0.5377
Frequent cough	1.17 0.73–1.89	1.21 0.76–1.93	0.9006
FEV_1 _< 80% of predicted value	3.79 1.64–8.74	1.35 0.66–2.77	0.0303
FVC <80% of predicted value	5.03 2.10–12.02	1.89 1.01–3.57	0.0445

Abbreviations:

FEV_1_: Forced expiratory volume in 1 second; FVC: Forced vital capacity;

### Respiratory health indicators as additional covariates for the association between air pollution exposure and cardiovascular mortality

In a previous paper we could provide evidence that an increase of exposure to PM_10 _was strongly associated with a reduction of lung function (FEV_1_: 5.1% (95% CI 2.5%–7.7%), FVC: 3.7% (95% CI 1.8%–5.5%)) as well as with increased frequency of respiratory symptoms [18]. In a further paper we have shown [19], that the association between air pollution levels and cardiopulmonary death rate was strong and statistically significant. This was also true for cardiovascular death, which we focused on in this paper (table 4). Table 4 shows the results of the Cox' regression analysis for the impact of air pollution exposure on cardiovascular mortality adjusted for confounders (model (a)) and additionally for respiratory disease or symptoms (model (b)). The risk ratios for the association between air pollution and cardiovascular mortality differed only marginally (<10%) between model (a) and model (b). We also tested all interactions between respiratory diagnosis and symptoms and air pollution on cardiovascular mortality. All p-values were above 0.3. Therefore no separate estimates in strata defined by respiratory health are given.

**Table 4:** The influence of respiratory health indicators (diagnoses and symptoms), assessed at baseline investigation, on the association between air pollution exposure (traffic, NO_2_, PM_10_) and cardiovascular mortality in a cohort of women aged 55 years at baseline investigation; results of a Cox' regression analysis.

	***<50 m distance to major road***			***NO***_2_***[16μg/m***^3^***] (five-year mean) ***^1^			***PM***_10_***[7μg/m***^3^***] (five-year mean)***^1^		
	***RR***	***95%-CI***	***p-value***	***RR***	***95%-CI***	***p-value***	***RR***	***95%-CI***	***p-value***
n/**N**		120/4457			97/4198			97/4198	

**Model (a)**, adjusted for potential confounders^3^	1.67	0.98–2.83	0.0573	1.72	1.24–2.39	0.0011	1.64	1.15–2.33	0.0056
**Model (b)**, additionally adjusted for									
Chronic Bronchitis by physician diagnose	1.63	0.96–2.76	0.0693	1.69	1.22–2.35	0.0017	1.62	1.14–2.30	0.0073
Frequent cough with phlegm production	1.71	1.01–2.88	0.0478	1.70	1.22–2.36	0.0015	1.62	1.14–2.31	0.0071
Frequent cough	1.71	1.01–2.88	0.0469	1.71	1.23–2.37	0.0013	1.63	1.15–2.32	0.0067

^1 ^Analyses on long term exposure to air pollution were made on subjects who were living longer than five years under their current address.

^2 ^Current smoking at the time of entering the study, no further adjustment for exposure to tobacco smoking

^3 ^Educational level and smoking

Abbreviations:

RR: Risk ratio; CI: Confidence interval; n/N: number of dead and sample size

Model (a)/(b): see text

For both lung function indicators the assumption of hazard proportionality over time was not valid. We therefore applied stratified Cox' regression analysis for model (b). The results are presented in table 5. In this sub-group of women with lung function measurements, the associations between traffic related pollution (NO_2 _and small distance to mayor road) and cardiovascular death were particularly strong. This again might be due to the study design which led to more pronounced contrasts in traffic related pollution. The associations between traffic related air pollution exposure (distance to major road and ambient NO_2_) and cardiovascular mortality were modified by impaired lung function. However, this modification was contrary to the meaningful expectation that impaired lung function would increase the risk ratio of air pollution exposure.

**Table 5:** The influence of lung function indicators, measured at baseline investigation, on the association between air pollution exposure (traffic, NO_2_, PM_10_) and cardiovascular mortality in a cohort of women aged 55 years at baseline investigation; results of a Cox' regression analysis.

	***<50 m distance to major road***			***NO***_2_***[16 μg/m***^2^***] (five-year mean) ***^1^			***PM***_10_***[7 μg/m***^2^***] (five-year mean) ***^1^		
	***RR***	***95%-CI***	***p-value***	***RR***	***95%-CI***	***p-value***	***RR***	***95%-CI***	***p-value***
n/N		52/2478			42/2328			42/2328	

**Model (a)**, adjusted for potential confounders^2^	2.33	1.09–4.95	0.0288	1.91	1.22–2.98	0.0048	1.26	0.75–2.14	0.3882
**Model (b)**, additionally estimated in strata defined by or adjusted^3 ^for:									
FEV_1 _< 80%				1.12	0.52–2.41	0.7683			
	2.27^4^	1.06–4.85	0.0339				1.14^4^	0.67–1.95	0.6352
FEV_1 _≥ 80%				2.23	1.27–3.89	0.0049			
FVC < 80%	1.21	0.28–5.25	0.7951	1.13	0.57–2.22	0.7329			
							1.13^4^	0.66–1.93	0.6621
FVC ≥ 80%	3.20	1.30–7.85	0.0112	2.38	1.30–4.34	0.0047			

^1 ^Analyses on long term exposure to air pollution were made on subjects who were living longer than five years under their current address.

^2 ^Educational level and smoking

^3 ^if p-value of interaction between air pollution exposure and lung function indicator was greater 0.3

^4 ^Common estimation for both strata because of no interaction between lung function indicator and air pollution exposure

Abbreviations:

RR: Risk ratio; CI: Confidence interval; n/N: number of dead and sample size; FEV_1_: Forced expiratory volume in 1 second; FVC: Forced vital capacity

Model (a)/(b): see text

## Discussion

Our study demonstrates that impaired respiratory health at the age of 55 is a risk factor for cardiovascular mortality. Women with impaired lung function had a higher cardiovascular mortality risk especially in the first years after the investigation. The impact of air pollution however was even less strong in these women than in those with normal lung function. We could not find an indication that women with impaired respiratory health would have an increased risk of suffering cardiovascular death associated with increased long-term exposure to air pollution. Therefore, long-term air pollution exposure and impaired respiratory health are independently associated with cardiovascular mortality.

Our findings in regards to the positive association between respiratory impairment and cardiovascular mortality are consistent with other published studies [23,8-10]. The studies from Schunemann et al. and Sin et al. also showed that decreased pulmonary function is a risk factor for cardiovascular mortality [8,10]. Yet, these studies did not investigate the relation between impaired respiratory health and air pollution-associated cardiovascular mortality. In contrast to these studies we found that the risk associated with impaired lung function declined over time.

There are several hypotheses about the general pathways of cardiovascular effects due to increased levels of air pollution [24,25]. One hypothesised that a biological pathway for cardiovascular mortality associated with long-term exposure to air pollution is pollution-induced lung damage. It suggests that in individuals who are susceptible, exposure to air pollution especially to ultrafine particles can induce alveolar inflammation, which subsequently result in respiratory illness and then in cardiovascular death [11,12]. The second hypothesis indicates that lung inflammation induced by air pollution not only leads to lung diseases, but independently can also cause vascular and heart diseases [25,26]. Alveolar macrophages and lung epithelial cells process inhaled particles or other air pollutants, this pro-inflammatory mediators not only promote a local inflammatory response in the lungs, but can also translocate into the circulation and induce a systemic inflammatory response [27]. Consequently, the possible biological pathway for this association is systemic inflammation and the progression of atherosclerosis [28]. Further, air pollution can lead to altered cardiac function due to a change in heart rate and blood pressure and finally lead to death [29-32].

The results of our study are more consistent with the second hypothesis. In fact, in our cohort study we could show that air pollution and impaired respiratory health are independently associated with cardiovascular death. Indeed women with already impaired lung function had a higher cardiovascular mortality risk especially in the first years after the investigation compared to those with normal lung function. But, increased levels of air pollution did not influence the mortality of these women. On the contrary, the relative risk of cardiovascular mortality associated with air pollution appeared to be higher in women without impaired lung function. In some women possibly, impaired lung function might be a sign for a still unknown but manifest cardiovascular disease which subsequently leads to early death not related to air pollution. However because of the relative small subgroups we chose a p-value of 0.3 to indicate an interaction. Therefore, the evidence for the variation in risk between the sub-groups is still not strong.

This observed result is in accordance with findings from the re-analysis of the Harvard Six City Study [4,5]. In their study, Krewski et al. reported about the risk of death associated with exposure to fine particles in different sub-groups among them those defined by lung function. In their study subjects with compromised lung function had a slightly greater risk of death. However, none of these interactions achieved statistical significance. The results of this re-analysis did not provide evidence of variation in risks among population sub-groups [5].

In a previous time series study, DeLeon et al. [14] observed that individuals with contributing respiratory conditions whose primary cause of death was circulatory were more affected by elevated levels of air pollution This role of respiratory disease in air pollution related cardiovascular mortality could not be confirmed in our study. There are two major differences to our study. First, the DeLeon-study focused primarily on daily mortality counts and the listing of the contributing respiratory causes on the death certificates. However, time-series studies can only investigate associations with the most recent exposure compared to cohort studies, which are able to show acute and chronic effects of air pollution on diseases and mortality. Second, DeLeon et al. demonstrated that the effect was only visible in older individuals (aged 75 and older) with underlying respiratory diseases. Older individuals were more susceptible to adverse effects of air pollution. The women followed up in our study were at most 73 years old. Therefore, the lack of effect in our study might be due to the younger age range.

Our study has certain limitations. The respiratory symptoms and the chronic bronchitis were self-reported, which might lead to some reporting bias. Furthermore, the women received only one lung function measurement, and we relied on cause-of-death data from death certificates which has the potential of bias for specific cause of death. As in most studies dealing with influences of covariates on survival of population groups, we chose Cox' Regression for analysis. This is basically a multiplicative approach. Therefore, our result of an independent association of air pollution and respiratory health on cardio vascular mortality can only be interpreted in this multiplicative context. The number of women with reduced lung function, respiratory diseases and cardiovascular mortality was low with respect to the statistical power of the study and was further reduced by stratification. Another limitation is the incompleteness of air pollution measurements. Values for the reference areas Borken and Dülmen before 1990 were imputed assuming similar trends as in the high-polluted areas. The estimation of ambient air PM_10 _concentrations by using TSP measurements may add another limitation to the study and may result into a bias of our risk ratio estimates. Indeed, assuming a smaller conversion factor for the rural area, for instance 0.65, which means greater fraction of coarse particles in TSP compared to the urban areas, the inconsistency of the results between table 4 and table 5 diminished. In tables 4 and 5, the risk ratios for PM_10 _using model (a) increased and showed similar results to the risk ratio for the influence of traffic and NO_2 _(data not shown). However, this modification of the TSP/PM_10 _conversion factor did not influence our main results, namely, the association between lung function and respiratory health indicators and cardiovascular mortality.

The strength of our analysis is the long follow-up of our cohort with multiple exposure assessments of air pollution levels and different respiratory health assessments (respiratory symptoms and lung function measurements).

In conclusion, the results from our analysis show that impaired respiratory health as measured by diagnoses, symptoms and lung function is related to an increased subsequent cardiovascular mortality. Women with impaired lung function had a higher cardiovascular mortality risk, especially in the first years after the investigation. We observed some indications that the impact of air pollution however was weaker in these women than in those with normal lung function. We therefore concluded that long-term exposure to high levels of air pollution affects respiratory health and cardiovascular death independently in a group of middle aged women. However, due to the short follow-up period of these women, we might have underestimated the long-term air pollution effects on less pronounced respiratory damage. A further follow-up study of these women is needed to provide more information about cardiovascular mortality in this group when they become older.

## Competing interests

The author(s) declare that they have no competing interests.

## Authors' contributions

TS performed the statistical and epidemiological analysis, drafted and wrote the paper. DS was co-investigator of the repeated cross-sectional studies, performed the Geographical Information System analysis, performed the statistical analysis and was responsible for the data management. UK was main investigator of the repeated cross-sectional studies, commented and advised on the statistical analysis and commented on the manuscript. UR was co-investigator of the repeated cross-sectional studies, commented and advised on the statistical analysis and commented on the manuscript. HEW commented on the manuscript. UG was co-investigator of the mortality follow-up and commented on the manuscript. JH was main investigator of the mortality follow-up and commented on the manuscript. All authors read and approved the final manuscript.
